# Extended cleavage specificities of mast cell proteases 1 and 2 from golden hamster: Classical chymase and an elastolytic protease comparable to rat and mouse MCP-5

**DOI:** 10.1371/journal.pone.0207826

**Published:** 2018-12-06

**Authors:** Michael Thorpe, Zhirong Fu, Emanuelle Albat, Srinivas Akula, Lawrence de Garavilla, Jukka Kervinen, Lars Hellman

**Affiliations:** 1 Department of Cell and Molecular Biology, Uppsala University, The Biomedical Center, Uppsala, Sweden; 2 GDL Pharmaceutical Consulting and Contracting, Downingtown, Pennsylvania, United States of America; 3 Tosoh Bioscience LLC, Separations Business Unit, King of Prussia, Pennsylvania, United States of America; Universität Stuttgart, GERMANY

## Abstract

Serine proteases constitute the major protein content of mast cell secretory granules. Here we present the extended cleavage specificity of two such proteases from the golden hamster, *Mesocricetus auratus*. Analysis by phage display technique showed that one of them (HAM1) is a classical chymase with a specificity similar to the human mast cell chymase. However, in contrast to the human chymase, it does not seem to have a particular preference for any of the three aromatic amino acids, Phe, Tyr and Trp, in the P1 position of substrates. HAM1 also efficiently cleaved after Leu similarly to human and many other mast cell chymases. We observed only a 3-fold lower cleavage activity on Leu compared to substrates with P1 aromatic amino acids. Chymotryptic enzymes seem to be characteristic for connective tissue mast cells in mammalian species from opossums to humans, which indicates a very central role of these enzymes in mast cell biology. HAM1 also seems to have the strongest preference for negatively charged amino acids in the P2´position of all mast cell chymases so far characterized. The second hamster chymase, HAM2, is an elastolytic in its activity, similarly to the α-chymases in rats and mice (rMCP-5 and mMCP-5, respectively). The presence of an α-chymase that developed elastase activity thereby seems to be a relatively early modification of the α-chymase within the rodent branch of the mammalian evolutionary tree.

## Introduction

Mast cells (MCs) are resident tissue cells frequently found in the connective tissue of the skin and around blood vessels and nerves as well as in the mucosa of the airways and intestines. They are therefore distributed primarily along both external and internal surfaces of the body, potentially acting as a first line of defence against incoming pathogens [1, 2]. To be able to exert its guardian function, MCs pre-store a number of potent inflammatory mediators in cytoplasmic granules. These granules are rapidly exocytosed following activation of the cell by IgE crosslinking or anaphylotoxin triggering. The majority of proteins found in these granules are serine proteases, which can be generally subdivided into chymases and tryptases [3–6]. Tryptases are tetrameric enzymes that cleave after basic amino acids i.e., arginine and lysine, whereas chymases are mostly chymotrypsin-like and cleave substrates after aromatic amino acids [7–9]. Phylogenetic analyses of the chymases have led to the identification of two distinct subfamilies, the α-chymases and the β-chymases [6, 10, 11]. The α-chymases are found as a single gene in all species investigated, except for ruminants, sheep and cattle, where two very similar α-chymase genes have been identified [6, 12]. The β-chymases have only been identified in rodents, dogs and cats [6]. Concerning the evolution of this locus, several additional major changes have occurred in rodents. For example, the rodent α-chymases, mouse mast cell protease (mMCP)-5 and rat mast cell protease (rMCP)-5, have changed primary cleavage specificity from aromatic amino acids (chymotrypsin-like) to aliphatic amino acids (elastase-like) [13–16]. Other subfamilies have also appeared in some mammalian lineages as exemplified by the mMCP-8 family in rodents and the duodenases in cows, sheep and pigs [6, 12]. This locus is therefore highly interesting, not only from an immunological view-point but also from an evolutionary perspective.

In order to study the appearance and diversification of these enzymes during vertebrate evolution we have analysed their genomic loci and determined the extended specificity of a number of mast cell chymases in various placental mammals including, dogs, mice, rats, humans and cynomolgus monkeys as well as in one marsupial, the American opossum [17–21]. All of them contain a classical chymase with chymotryptic activity, however with slightly different primary and extended specificities. A majority also show a relatively strong preference for negatively charged residues in the P2´position of the substrate, as well as dominance of aliphatic amino acids both upstream and downstream of the cleavage site. We have previously shown that the P2´preference for negatively charged amino acids is, in the human chymase, primarily dependent on two enzyme residues: Arg143 and Lys192 [22]. Mutational analysis shows that both are important, where each contributes approximately 50% towards the preference effect [22].

The primary and extended specificities of these enzymes are of key importance for their target specificity and therefore their biological function. Determination of these key characteristics of these enzymes from a panel of different mammalian species can thereby give us clues to their conserved biological functions. One of the key questions in the context of rodent mast cell enzymes, and in particular their chymases, is when the α-chymase mutated into a potent and highly specific elastase in its catalytic specificity. Furthermore, with this knowledge, what is the function of this new specificity in rodent mast cells? This change in specificity of the α-chymases is also directly connected to the appearance of the β-chymases. Here, in both mice and rats, they have taken over the chymase function from the mutated α-chymase. Their β-chymases, mMCP-4 and rMCP-1, have become the primary chymotryptic enzymes when the α-chymases became elastases [18]. This is a strong indication that the chymase is of central importance for the biological function of the mast cell. Another interesting question is the evolutionary timing of the appearance of the mMCP-8 family of chymase-locus encoded proteases. mMCP-8 is the first identified basophil-specific gene in any species and has therefore attracted attention as both a basophil specific marker as well as a gene to produce basophil ablated mice for in depth studies of basophil biology [23–25].

**Fig 1:**
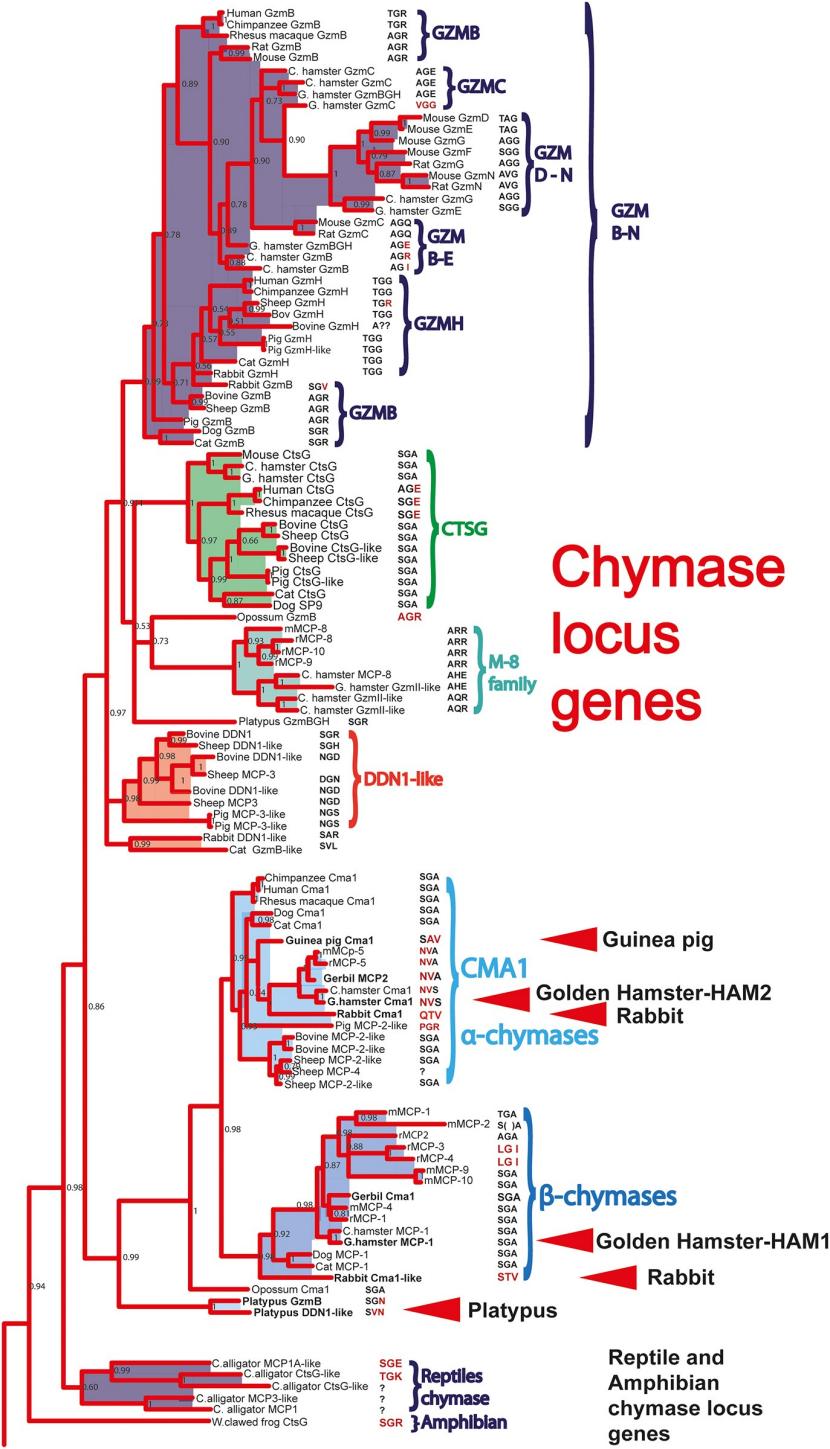
Phylogenetic tree of serine proteases encoded from the chymase locus in vertebrate species. A panel of vertebrate chymase locus encoded genes were analyzed for their sequence relatedness using the MrBase analysis program and the Maximum-likelihood algorithm [6]. The major enzyme clusters are marked by brackets and their names. The genes of particular interest for this study are marked with red arrows.

Initial studies using chromogenic substrates and X-ray crystallography analyses show that the hamster chymase 1 (HAM1) is a typical chymase with selectivity for the aromatic amino acids Tyr and Phe but also Leu, whereas hamster chymase 2 (HAM2) is an elastase with preference for the aliphatic amino acids Ala and Val in the P1 position of substrates [15]. However, no detailed analyses of their extended specificities have been performed so far. In order to address this question, here we have determined the extended specificities of these two serine proteases from the golden hamster (*Mesocricetus auratus)* by phage display technique. Furthermore, we have performed a detailed quantitative analysis of the importance of individual amino acids at, and around, the cleavage site for the efficiency in cleavage by the use of a panel of recombinant substrates. We have also studied the genomic organization of the chymase locus in a panel of rodents. Our results confirmed that the hamster α-chymase (HAM2) is an elastase, thereby indicating an early change in specificity in the rodent branch of the mammalian evolutionary tree, and that one β-chymase has taken a role as the primary chymotryptic enzyme. Interestingly, this hamster enzyme (HAM1) is also the mammalian chymase with the strongest P2´preference for negatively charged amino acids. Based on the phylogenetic tree (Fig 1) we also conclude that the golden hamster and the Chinese hamster both have mMCP-8 related genes, also indicating that these enzymes appeared early in rodents. This gene has not been found in any other mammalian species except rodents, indicating rodent-specific functions for both the mMCP-8 related proteases as well as elastase specific α-chymases.

## Results

### Phylogenetic analysis

A phylogenetic analysis has been performed to determine the relatedness in primary structure of the two hamster proteases to other mammalian mast cell proteases. This analysis is a part of a larger analysis of the evolution of the hematopoietic serine proteases presented in Akula et al 2015 where in total 368 different hematopoietic serine proteases were analyzed by several different algorithms [6]. Four additional trees are, in addition to the tree in this article, found in the supplementary material of that article [6]. As can be seen from Fig 1 HAM1 cluster firmly with the other rodent β-chymases whereas the HAM2 cluster with the other rodent α-chymases. Two rabbit chymases and a guinea pig enzyme, which will be discussed later in this communication as interesting representatives of proteases from an early branching rodents, does in this analysis cluster outside the tight inner cluster of the rodent α-and β-chymases (Fig 1) [6].

### Purification and activation of recombinant HAM1 and HAM2

**Fig 2:**
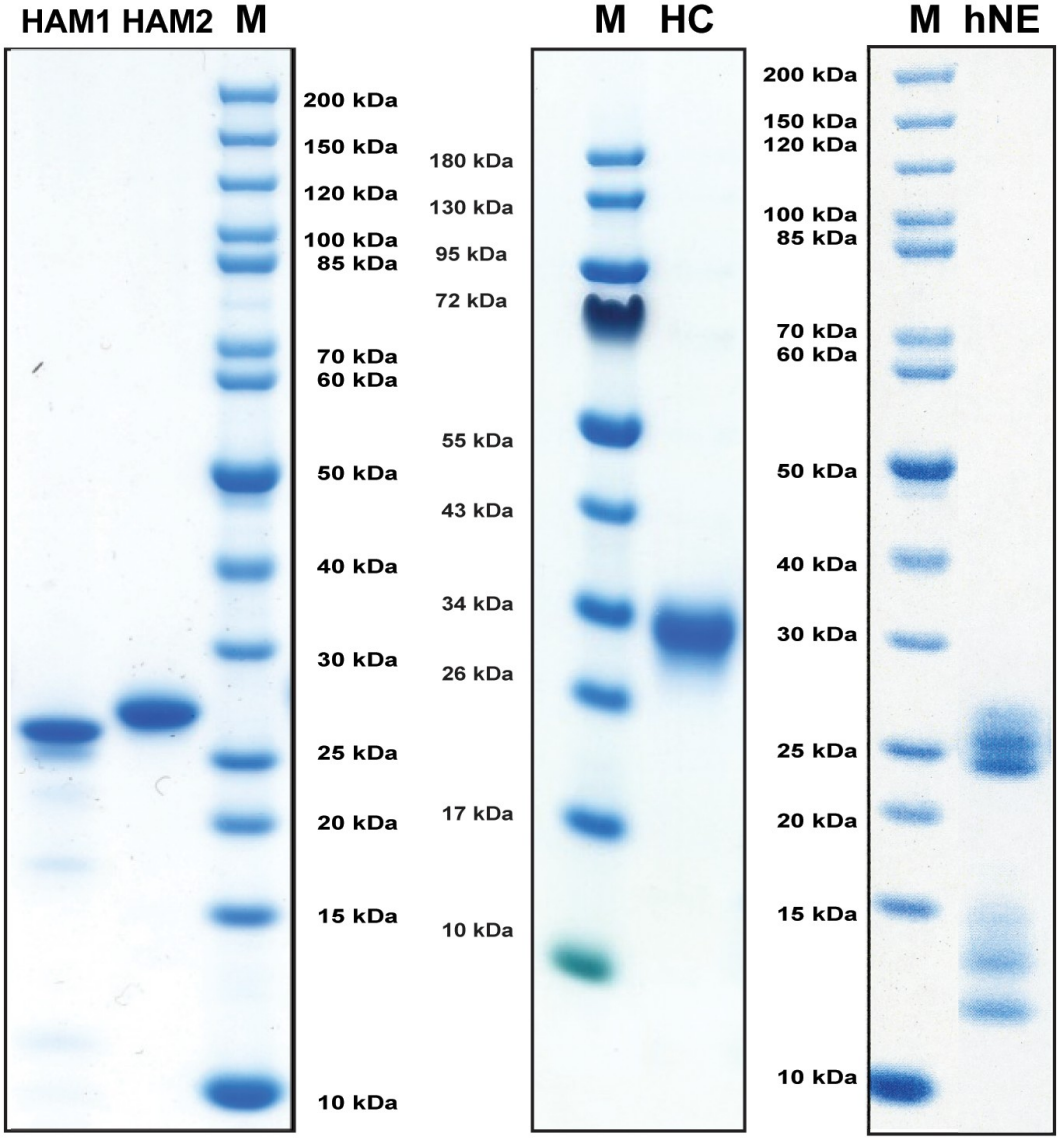
SDS-PAGE of purified chymases and related reference proteases used in this study. Three different recombinant mast cell chymases were expressed in a baculovirus expression system. The purified active enzymes were analyzed by separation on SDS-PAGE and visualized with Coomassie Brilliant Blue staining. Proteases: HC, human chymase; hNE, human neutrophil elastase; HAM 1 and 2, hamster chymases 1 and 2. M is molecular weight marker.

The coding regions for the golden hamster HAM1 and HAM2 were inserted into the baculovirus vector pAcGP67B. The three chymases were expressed in baculovirus-infected insect cells and purified as previously described [15]. Following purification, the fully mature chymases (25–28 kDa) were ≥95% pure as determined by SDS-PAGE (Fig 2) and the correct N-termini were confirmed by N-terminal sequencing. Mass spectral analysis has suggested that HAM2 is heterogeneously glycosylated, but no glycan side chains were attached to HAM1 [15]. Recombinant human chymase, produced in baculovirus infected insect cells as described above and a commercial preparation of human neutrophil elastase (Fig 2), were used as reference enzymes in the chromogenic substrate assays.

### Chromogenic substrate assays

**Fig 3:**
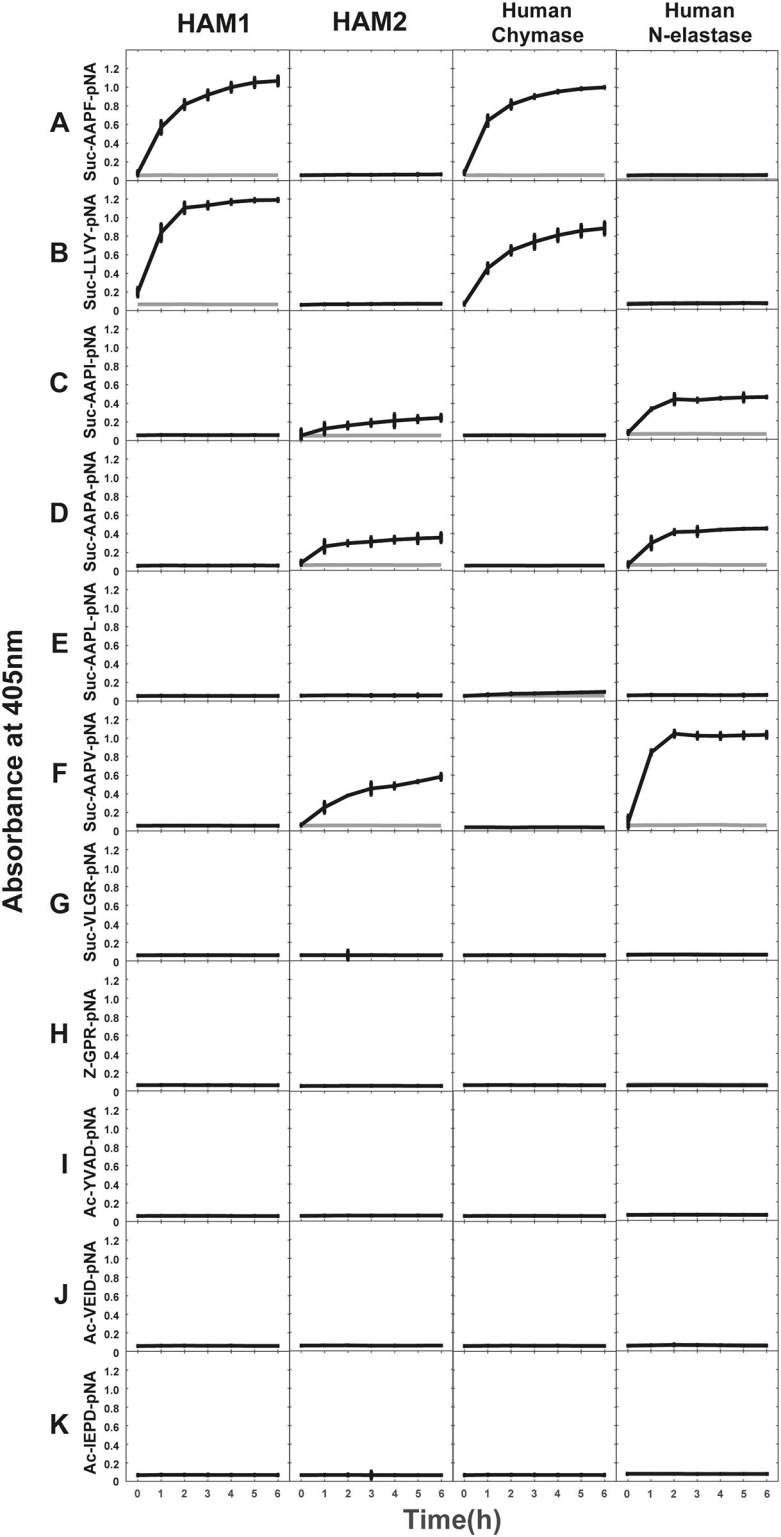
Chromogenic substrate assays. A panel of chromogenic substrates was used to determine the primary specificity of HAM1 and HAM2. Specificities of the human chymase and human neutrophil elastase are presented as reference. The panel includes different chymase, elastase, tryptase and aspase substrates. The amino acid sequences of the substrates are listed at the left side of the panels. The analyses were done in triplicates and the standard deviation within these triplicates is presented in the figure.

A panel of chromogenic substrates were used to determine the primary specificities of the two hamster chymases. The panel included chymase, elastase, tryptase and asp-ase substrates. HAM1 cleaved the substrates with Phe and Tyr very efficiently but not any of the other substrates (Fig 3, column 1) that is consistent with known human chymase specificity (Fig 3, column 3). In contrast, HAM2 did not cleave any of the chymase substrates but instead effectively cleaved the elastase substrates with a Val, Ala or an Ile in the P1 position (Fig 3, column 2). HAM2 thereby showed almost the same characteristics as the human neutrophil elastase that was included as a reference (Fig 3, column 4). Interestingly, no cleavage was observed for the Leu containing substrates for any of the hamster chymases nor the human chymase.

### Determination of the extended cleavage specificity by substrate phage display

To determine the extended cleavage specificities of the two hamster mast cell proteases a phage T7 based system was used where individual peptide sequences are displayed on the surface of the phage. The phage library used contained approximately 5x10^7^ phage clones. Each phage clone expresses a unique sequence of 9 random amino acids (nonamer), which enables the characterization of a region covering both 4–5 amino acids upstream and downstream of the cleavage site. The random region in each phage clone is followed by a His_6_-tag at the C-terminus of capsid protein 10. The phages can thereby be immobilized on Ni-NTA agarose beads. Selections of nonamers susceptible to cleavage by the two proteases were performed over 8 and 5 biopannings (HAM1 and HAM2, respectively), after which they induced the release of 15 and 107 times more phages compared to a PBS control, respectively (data not shown).

After the last biopanning, 120 individual phage clones were isolated for each of the two proteases, and the region containing the nine amino acids random region was amplified by PCR. 96 of these PCR samples with, strong and clean PCR fragments were sent for sequencing. The sequences encoding the randomly synthesized nona-peptides were then translated into nona-peptides, which were aligned based on the results from the chromogenic substrate assay for P1 specificity and for similarities to the cleavage specificity of the human chymase and mMCP-4 for HAM1, and rMCP-5 for HAM2 [14, 19].

**Fig 4:**
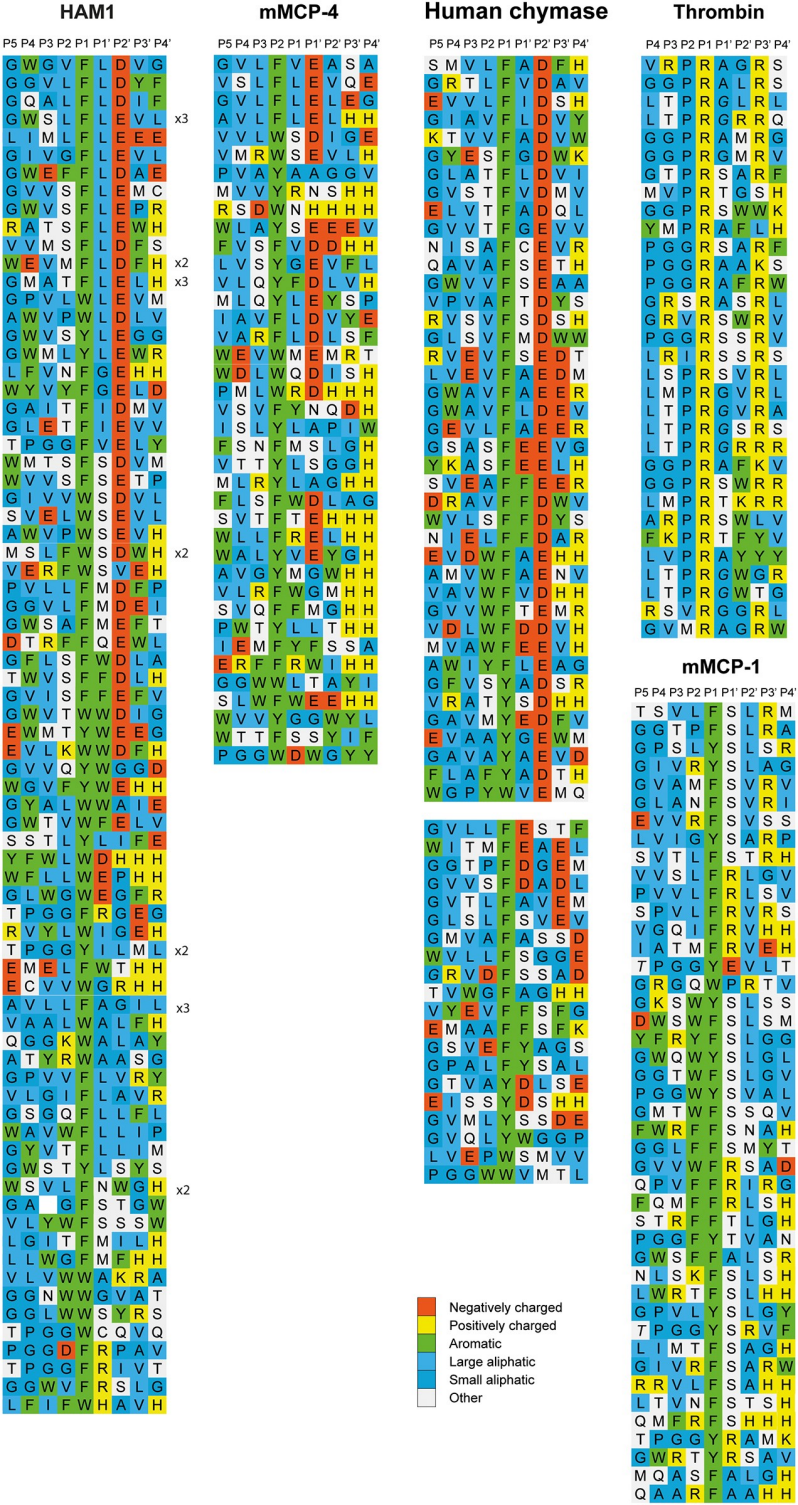
Phage displayed nonamers susceptible to cleavage by HAM1 after five biopannings. After the last selection step, phages released by proteolytic cleavage of the three proteases were isolated and the sequences encoding the nonamers were determined. The general sequence of the T7 phage capsid proteins are PGG(X)_9_HHHHHH, where (X)_9_ indicates the randomized nonamers. The protein sequences were aligned into a P5-P4´ consensus, where cleavage occurs between positions P1 and P1´. If the sequence was found more than once this is indicated by the corresponding number to the right of the sequence. The amino acids are colour coded according to the side chain properties as indicated in the legend. For comparison phage display data from four additional enzymes were included in the figure; mMCP-4, human chymase, human thrombin and mMCP-1 [17–19, 27].

**Fig 5:**
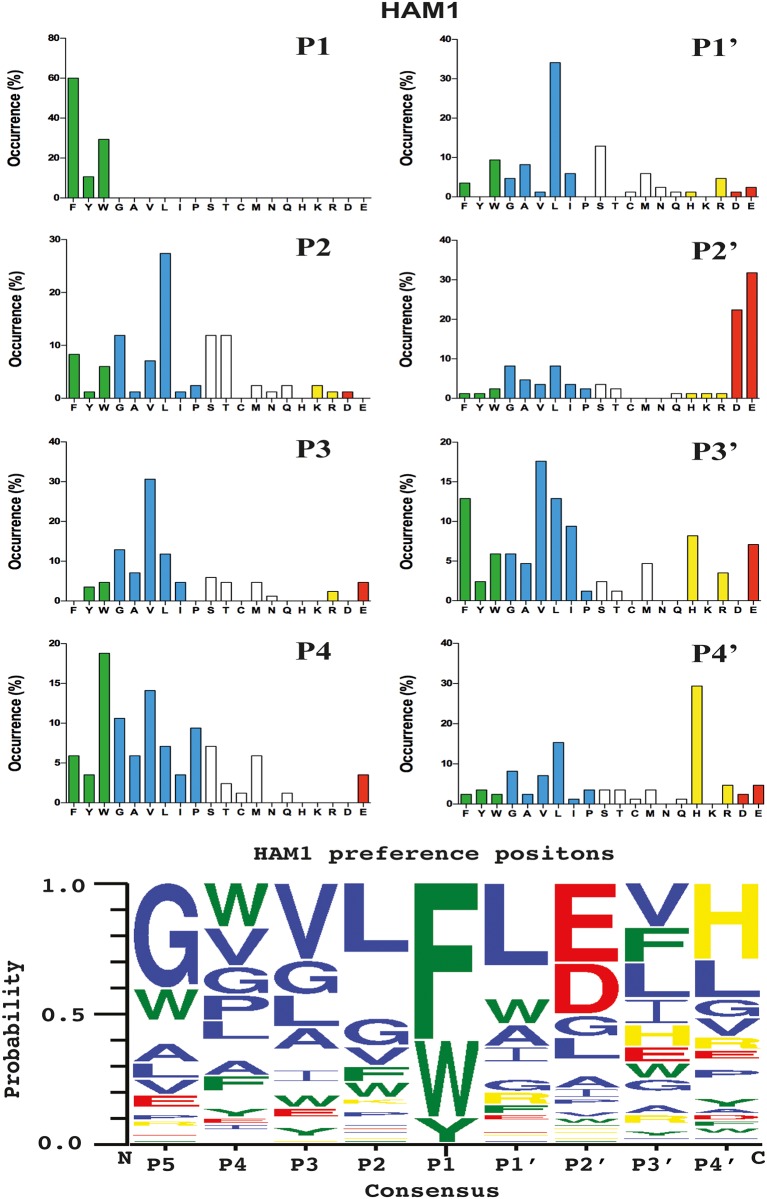
Distribution of amino acids in positions P4 to P4´ in phage displayed nonamers cleaved by HAM1 after five biopannings. Based on the alignment in Fig 4 the percentage of each amino acid present in each position P4 to P4´ as calculated. The amino acids are ordered from left to right: aromatic, aliphatic, hydrophilic, basic (positively charged) and acidic (negatively charged). In the bottom of the figure we have used the computer program WebLogo to generate an illustration of the relative distribution of the amino acid preferences for cleavage by HAM1 as obtained from the phage display analysis.

HAM1 showed a very strict chymotryptic activity with a preference for aromatic amino acids in the P1 position (Figs 4 and 5). A relatively equal distribution for the three aromatic amino acids, Phe, Tyr and Trp was observed in the P1 position, with also a high frequency of aliphatic amino acids both upstream and downstream of the cleavage site. A strong preference for negatively charged amino acids in the P2´position, similar to what is seen for the mouse and human chymases, was also observed (Figs 4 and 5).

**Fig 6:**
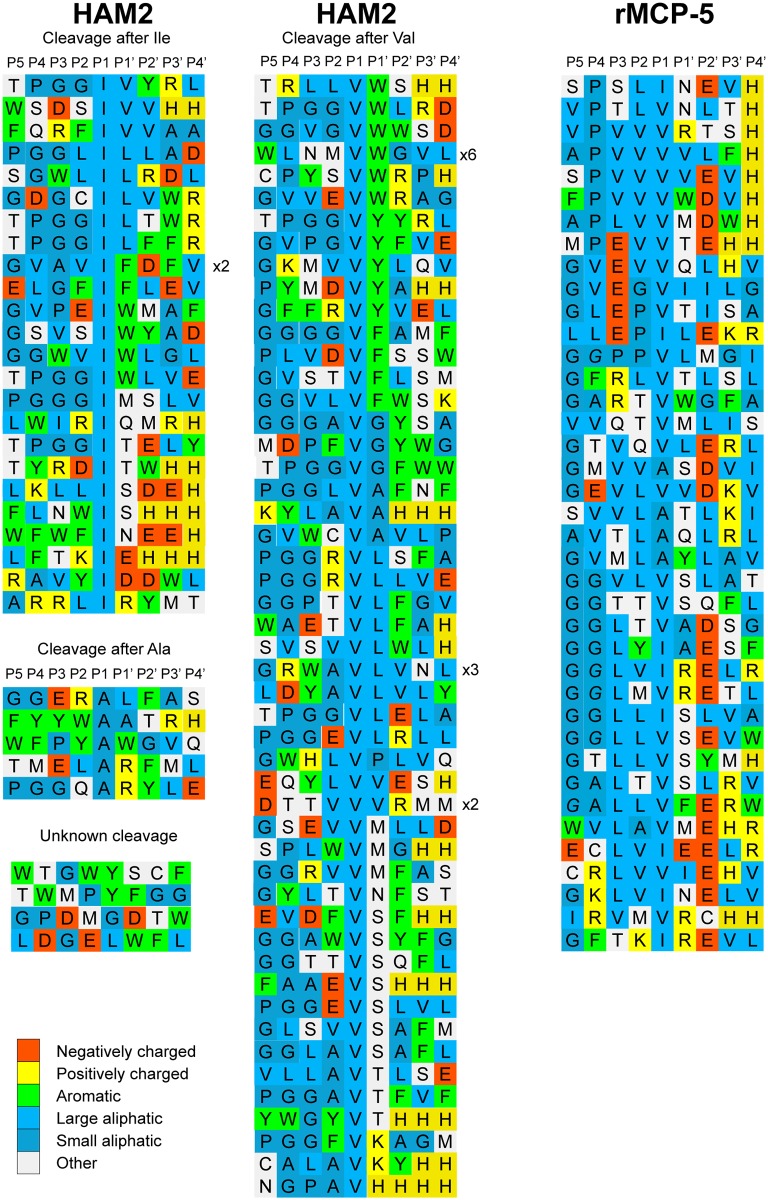
Phage displayed nonamers susceptible to cleavage by HAM2 after eight biopannings. After the last selection step, phages released by proteolytic cleavage of the three proteases were isolated and the sequences encoding the nonamers were determined. The general sequence of the T7 phage capsid proteins are PGG(X)_9_HHHHHH, where (X)_9_ indicates the randomized nonamers. The protein sequences were aligned into a P5-P4´ consensus, where cleavage occurs between positions P1 and P1´. If the sequence was found more than once this is indicated by the corresponding number to the right of the sequence. The amino acids are colour coded according to the side chain properties as indicated in the legend. For comparison, phage display data for another elastase specific chymase, rMCP-5 [14], is included in the figure.

**Fig 7:**
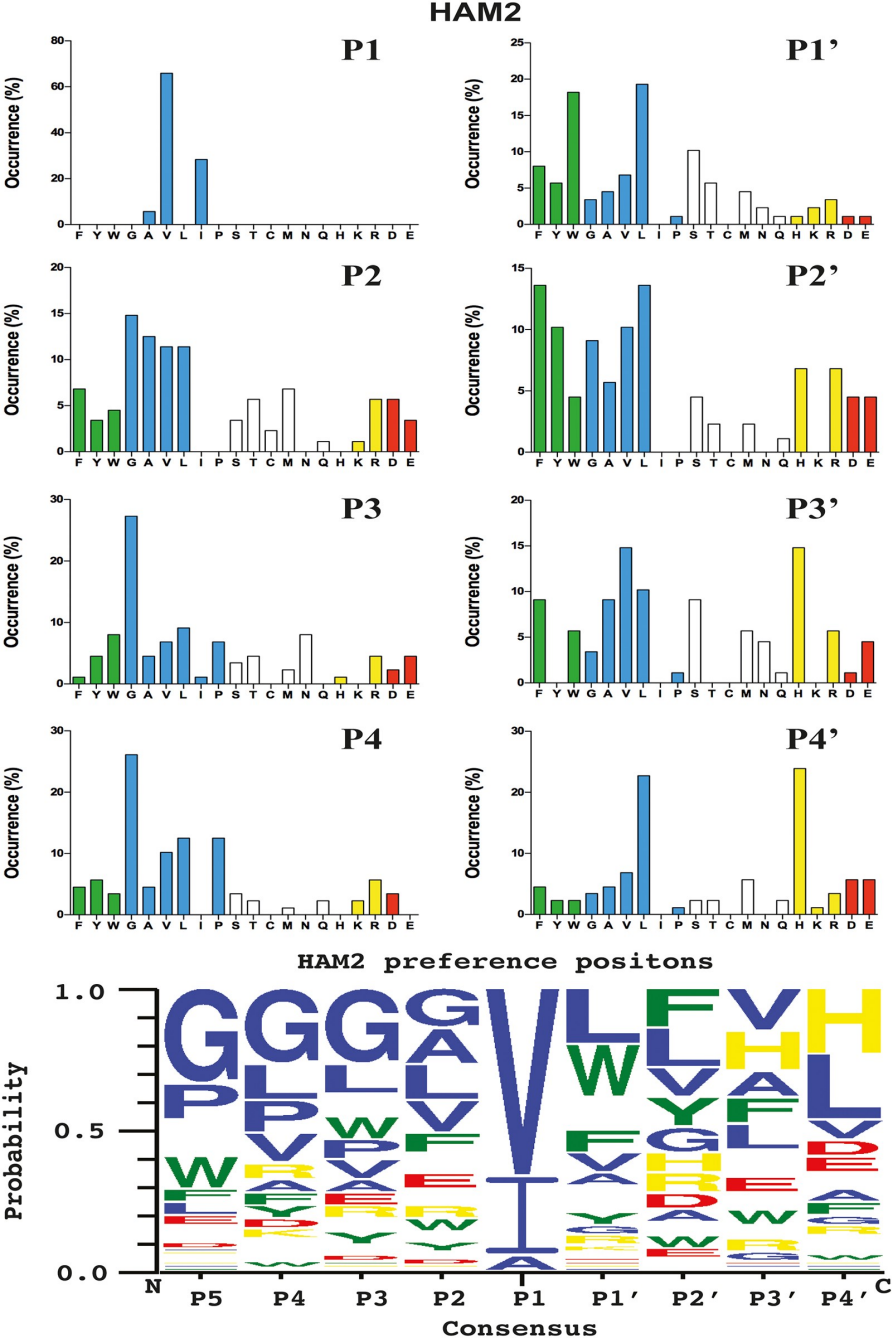
Distribution of amino acids in positions P4 to P4´ in phage displayed nonamers cleaved by HAM2 after five biopannings. Based on the alignment in Fig 6 the percentage of each amino acid present in each position P4 to P4´ as calculated. The amino acids are ordered from left to right: aromatic, aliphatic, hydrophilic, basic (positively charged) and acidic (negatively charged). In the bottom of the figure we have used the computer program WebLogo to generate an illustration of the relative distribution of the amino acid preferences for cleavage by HAM2 as obtained from the phage display analysis.

When analysing the phage clones for HAM2, a high number of aliphatic amino acids were observed (Figs 6 and 7). Based on the results from the chromogenic substrate assay, the alignment was assigned to give preference for the three aliphatic amino acids Val, Ile and Ala in the P1 position (Fig 6). A marked overrepresentation of Val and Ile over Ala in the P1 position was observed as well as positions surrounding the cleavage site with a marked overrepresentation of aliphatic and aromatic amino acids both upstream and downstream of the cleavage site. There was also a slight preference for uncharged hydrophilic amino acids, including Ser, Thr and Met in the P1´position (Figs 6 and 7). However, no marked overrepresentation of negatively charged amino acids in the P2´ was observed, which is different from observations from the rat homologue, rMCP-5 (Fig 6).

### Verifying the consensus sequence by the use of recombinant protein substrates

**Fig 8:**
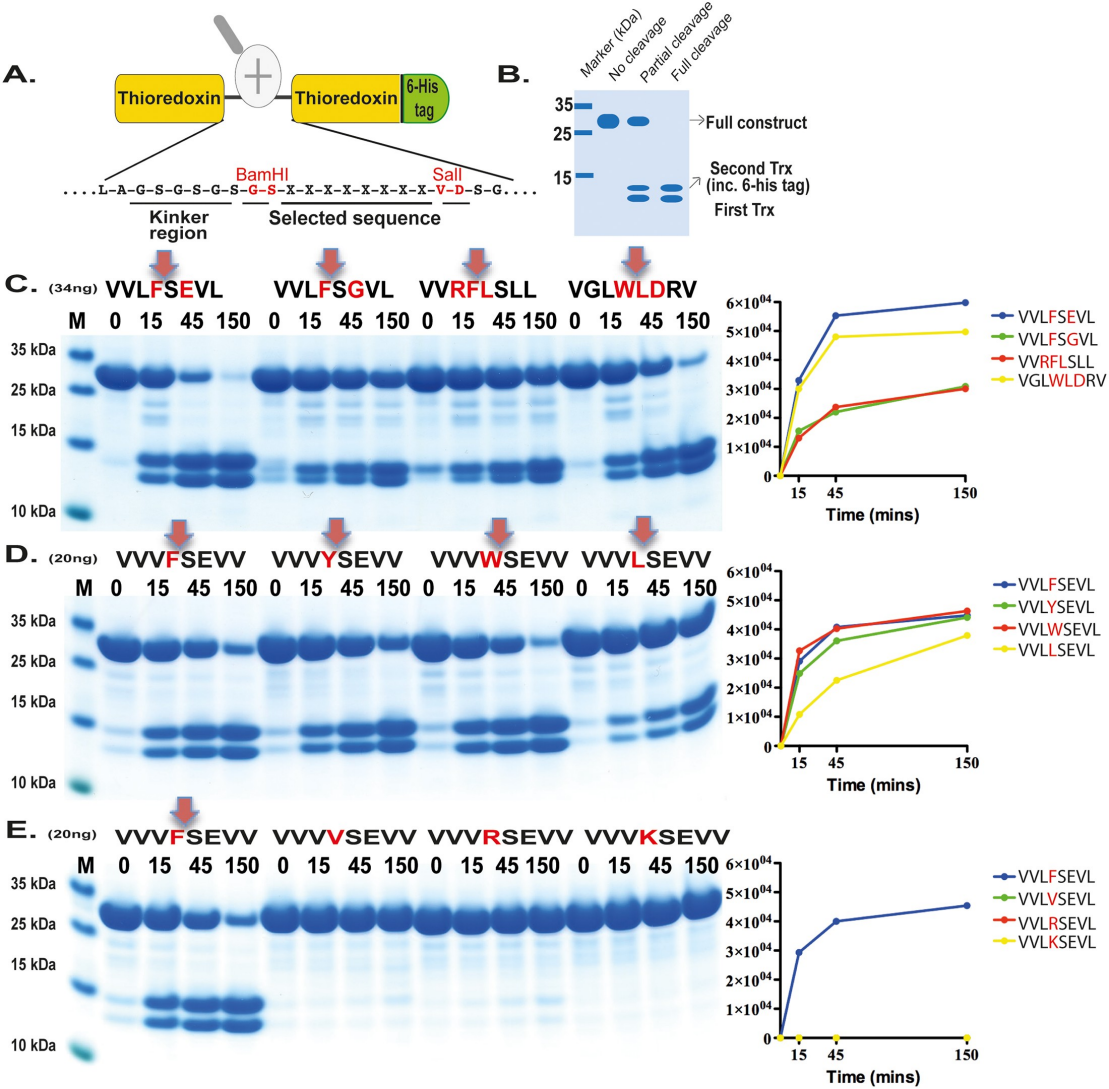
Analysis of the cleavage specificity of HAM1 by the use of recombinant protein substrates. Panel A shows the overall structure of the recombinant protein substrates used for analysis of the efficiency in cleavage by HAM1. In these substrates two thioredoxin molecules are positioned in tandem and the proteins have a His_6_-tag positioned in their C termini. The different cleavable sequences are inserted in the linker region between the two thioredoxin molecules by the use of two unique restriction sites, one Bam HI and one SalI site, which are indicated in the bottom of panel A. In panel B an example cleavage is shown to highlight possible cleavage patterns. Panels C, D and E shows the cleavage of a number of substrates by HAM1. The sequence of the different substrates are indicated above the pictures of the gels. The time of cleavage in minutes is also indicated above the corresponding lanes of the different gels. The uncleaved substrates have a molecular weight of approximately 25 kDa and the cleaved substrates appear as two closely located bands with a size of 12–13 kDa.

**Fig 9:**
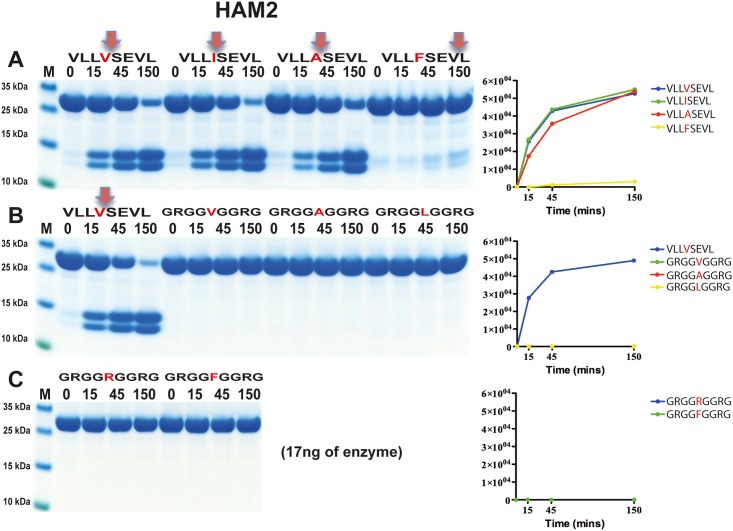
Analysis of the cleavage specificity of HAM2 by the use of recombinant protein substrates. Substrate sequences are indicated above the gel images. The time of cleavage in minutes is indicated above the corresponding lanes of the different gels. The uncleaved substrates have a molecular weight of ~25 kDa and the cleaved substrates appear as two close bands with a size of 12–13 kDa.

In order to validate the phage display sequence data and to address small variations of amino acids in the aligned phages a system was developed where by a number of sequences were analysed by the cleavage of recombinant substrates in a two-thioredoxin (trx) system (Fig 8A). These recombinant protein substrates, have been used in a number of previous studies and there been shown to be highly reliable and useful for the validation of the results from the phage display [20, 22, 26–30]. In this system double stranded oligonucleotide encoding the actual sequence is inserted in the linker region between two *E*.*coli* thioredoxin (trx) proteins by ligating into a BamHI and a SalI site of the vector construct (Fig 8A). For purification purposes a His_6_-tag was added to the C-terminal of this protein (Fig 8A). We usually start with the consensus sequence obtained from the phage display analyses followed by a number of related and unrelated substrate sequences to obtain a more complete picture of the selectivity of the enzyme under analysis. All of these substrates were expressed as soluble proteins in *E*.*coli* and purified on IMAC columns to obtain a protein with a purity of 90–95%. These recombinant proteins were then used to study the preference of the two hamster chymases for these different sequences (Figs 8 and 9).

The analysis of HAM1 confirmed the finding from the phage display analysis that this protease had a relatively strong preference for negatively charged amino acids in the P2’ position, similar to what has been observed for many other mammalian mast cell chymases including the human chymase and mMCP-4 (Fig 8C) [19]. It has what is probably the strongest preference for negatively charged amino acids in this position that we have seen among all the mast cell chymases analysed so far. We also saw relatively small differences between the three aromatic amino acids in the P1 position, which also reflected the results from the phage display analysis. Both Phe and Trp substrates were cleaved with almost equal efficacy and only a slightly lower activity was observed on the Tyr containing substrate (Fig 8D). Like many other mammalian mast cell chymases, substrates with Leu in the P1 position were also cleaved relatively efficiently. We observed a drop in activity by a factor 3 of the Leu substrate compared to the most efficiently cleaved substrates having Phe or Trp in the P1 position (Fig 8D). No cleavage or only very minor was observed for the substrates containing a Val, Arg or Lys in the P1 position, showing that this enzyme is a strict chymase (Fig 8E).

HAM2 showed a very different cleavage preference (Fig 9) compared to HAM1, which was also seen in the phage display analysis. Here, no activity against the Phe containing substrate was seen, instead it effectively cleaved the three substrates having an aliphatic amino acid in the P1 position (Fig 9A). The substrates with a Val, Ile or Ala in the P1 position were cleaved almost equal catalytic rate, with possibly a slight preference for Val and Ile over Ala (Fig 9A). There was also a strong dependence on surrounding residues next to the cleavage site. Small amino acids such as Gly were not favoured in the near vicinity of the cleavage site (Fig 9B and 9C). This pattern was not consistent with the result from the phage display sequences (Fig 6). However, many of the Gly residues upstream of the cleavage site in the phage display sequences originate from the phage arm which ends with PGG, which result in an overrepresentation of Gly residues within this region of the phage display sequences. Interestingly, from the phage display we saw a higher number of aromatic amino acids in the surrounding of the cleavage site compared to what was observed for rMCP-5 (Fig 6).

## Discussion

Compared to most other placental mammals, several major changes in the repertoire of the different mast cell chymotryptic enzymes have occurred in rodents. One of the most significant is the change in primary specificity of the α-chymase, where rodent α-chymases have become elastases in their catalytic specificity. The second is the appearance of two new subfamilies of enzymes, one that is closely related to the original α-chymase, and one completely new family more related to cathepsin G and the granzymes. The second family has been named the mMCP-8 family from the first gene of that family identified, the mMCP-8 gene in the mouse [23, 24]. The first of the two new families, the new α-chymase related genes are the β-chymases. They are located downstream of the α-chymase within the chymase locus [5, 6]. In both mice and rats, this region has undergone a massive expansion: There are at least four transcriptionally active β-chymases in the mouse and five in the rat [5, 6]. One of the questions we have addressed in this study is the evolutionary timing of these two events. We can now, from the analyses of the two hamster enzymes, say that the mutations resulting in the change in primary specificity of the α-chymase occurred relatively early in the rodent lineage. Interestingly, in all three rodent species analysed so far (rats, mice, golden hamster) where the chymotryptic activity of the α-chymase has been lost, there the β-chymases have taken over the role as the major chymotryptic mast cell enzymes. Together with the presence of highly proteolytically active chymotryptic enzymes in all mammalian species analysed so far, this gives very strong indications that a chymotryptic enzyme is a key characteristic of mammalian connective tissue mast cells.

**Fig 10:**
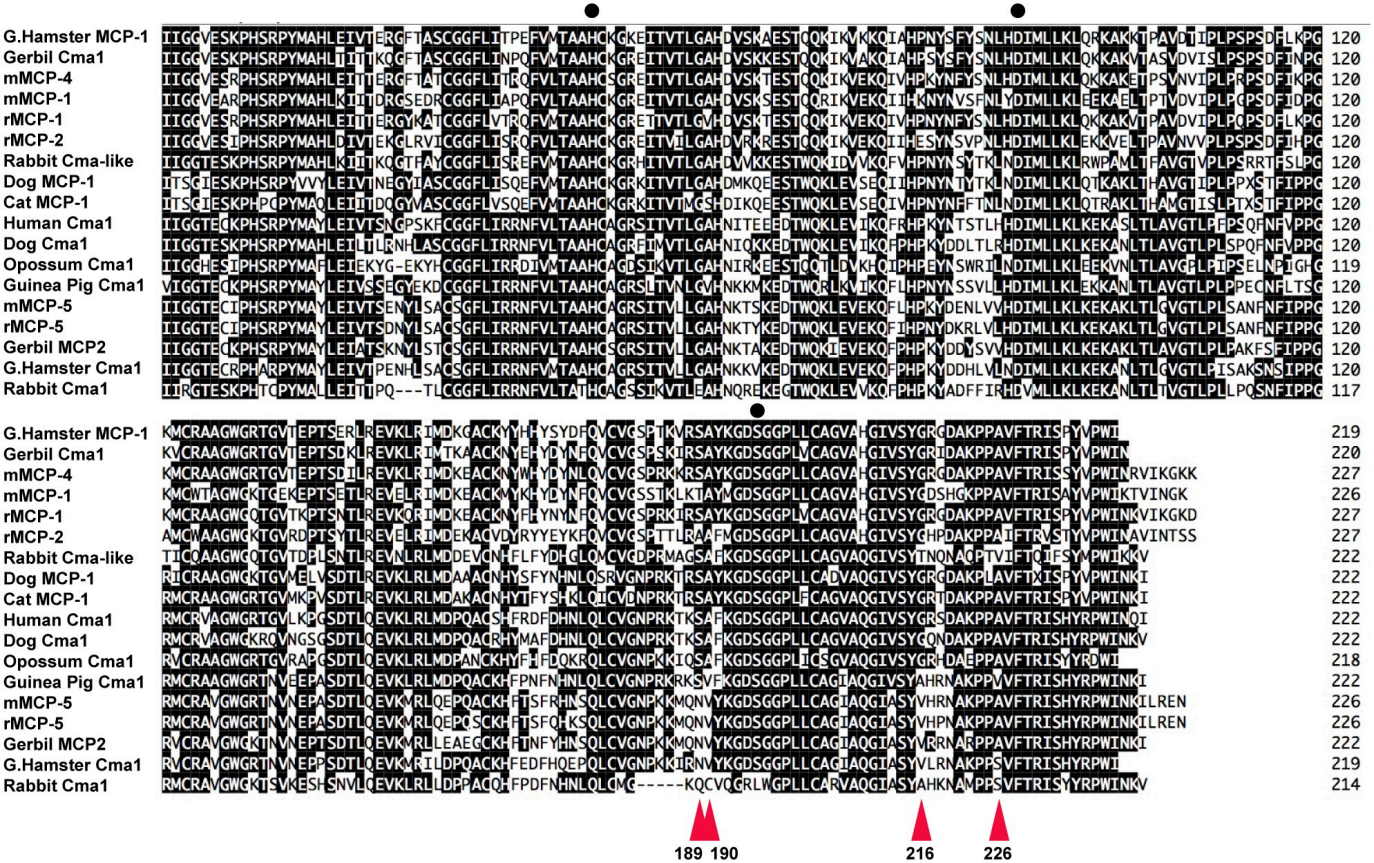
Alignment of a number of mammalian alpha and beta chymases. A panel of mammalian alpha and beta chymases were aligned using the DNA Star Megalign program and using the Clustal W algorithm. The positions of the three residues of the catalytic triad His-Asp-Ser are marked by black dots. The positions of the four amino acids of the substrate pocket, residues 189, 190, 216 and 226, are marked by red arrows. These four residues are for many of these proteases (at least the mammalian enzymes) of major importance for their P1 selectivity [5, 6, 15, 16].

To further search for the origin of the mutations that resulted in a major shift in primary specificity of the α-chymase, one species is of particular interest, the rabbit. Rabbits may represent a very early branching point of the rodent lineage, which was also indicated from the phylogenetic tree (Fig 1). However, when comparing the sequences of the two rabbit enzymes that in the phylogenetic tree, clustered with the α− and β-chymases respectively, a few interesting features of these two enzymes were found (Fig 10). In the alignment, the rabbit α-chymase (Rabbit Cma-like in Fig 10) appears to have been inactivated by mutations affecting both the active site as well as the N-terminal hydrophobic region involved in the activation of the protease (Fig 10). The Ser of the catalytic triad has been mutated into a Leu and a charged amino acid, an Arg, has been introduced into the four amino acids N-terminal region, which is usually hydrophobic and often having the characteristic sequence Ile-Ile-Gly-Gly. These two mutations most likely turn the enzyme into a non-functional pseudogene (Fig 10). The second rabbit enzyme (Rabbit Cma1 in Fig 10), which clusters closely with the β-chymases also had acquired a number of mutations that has affected the substrate pocket. These mutations may have effects on both its primary and extended specificities. In the positions 189, 190, 216 and 226 (chymotrypsinogen numbering), which are, based on X-ray crystallography analysis of HAM2 [15], known to affect the P1 specificity due to their position in lining the active site pocket, is in all the active chymases (SAGA, or TAGA or AAGA) (Fig 10). The elastases mMCP-5, rMCP-5 and HAM2 instead have NVVA or NVVS in these positions. Interestingly, the rabbit CMA1-like gene, which is the β-chymase homologue, instead has the amino acids SATV forming its substrate binding pocket, indicating a more narrow pocket (especially affected by Thr instead of Gly in the position 216) compared to the classical chymases (Fig 10). However, no kinetic data are available yet regarding these rabbit enzymes, therefore the answers to their activity and cleavage specificities needs to wait for more detailed analyses of these interesting enzymes.

The appearance of the β-chymases in mammals is also still not fully resolved. We can see that in all rodents studied, including rats, mice and hamsters, all contain β-chymases, and furthermore, they have taken over the primary chymase activity from the α-chymases. Interestingly, the guinea pig has been shown to have Leu specificity [16]. Despite this, the picture of the β-chymase in rodents is therefore relatively clear, perhaps with the guinea pig and rabbit as exceptions, as previously described. However, for the cat and dog enzymes, which cluster with the rodent β-chymases, the picture is much less clear (Figs 1 and 10). In several updates of the dog genome, the potential dog β-chymase has changed significantly, from being a pseudogene, into a functional gene. Another gene in the locus that was present but is now lost in this update is granzyme H. Subsequently, in the latest genome update, granzyme H is back and the β-chymase is now no longer there. This highlights the genome sequences are still not fully completed and a lot of caution needs to be taken when analysing the evolution of these proteases, not only in this locus but also with other genes and gene loci of presumed completed genomes. The β-chymase genes from the previous genome assemblies also contained a region of undefined nucleotides, which in both dog and cat sequences are located in the presumed coding region. Therefore the gene is most likely there despite the fact that it is not found in the last genome update. In addition, to our knowledge no recombinant protein has been produced for these β-chymases, which is why we do not know if they are functional, and in that case what specificity they have. This was the case for the rabbit α-chymase and also the dog and cat β-chymases, which have a somewhat peculiar N-terminal region including two hydrophobic residues (if they exist) (Fig 10). Instead of a classical Ile-Ile-Gly-Gly motif, they both have Ile-Thr-Ser-Gly, indicating they may have problems being fully active after removal of the activation peptide (Fig 10). The sequence from an early assembly also contained a frame shift in the sequence of exon 5, and by PCR we amplified this region from wolves to see if the presumed inactivation was dog specific. We found that this frame shift also was also present in wolves, indicating an early inactivation of this gene [5]. Therefore several unanswered questions remain concerning these presumed β-chymases in dogs and cats. We have also failed in several attempts to isolate the cDNA by PCR amplification from dog tissues, also indicating that the gene may be transcriptionally inactive.

**Fig 11:**
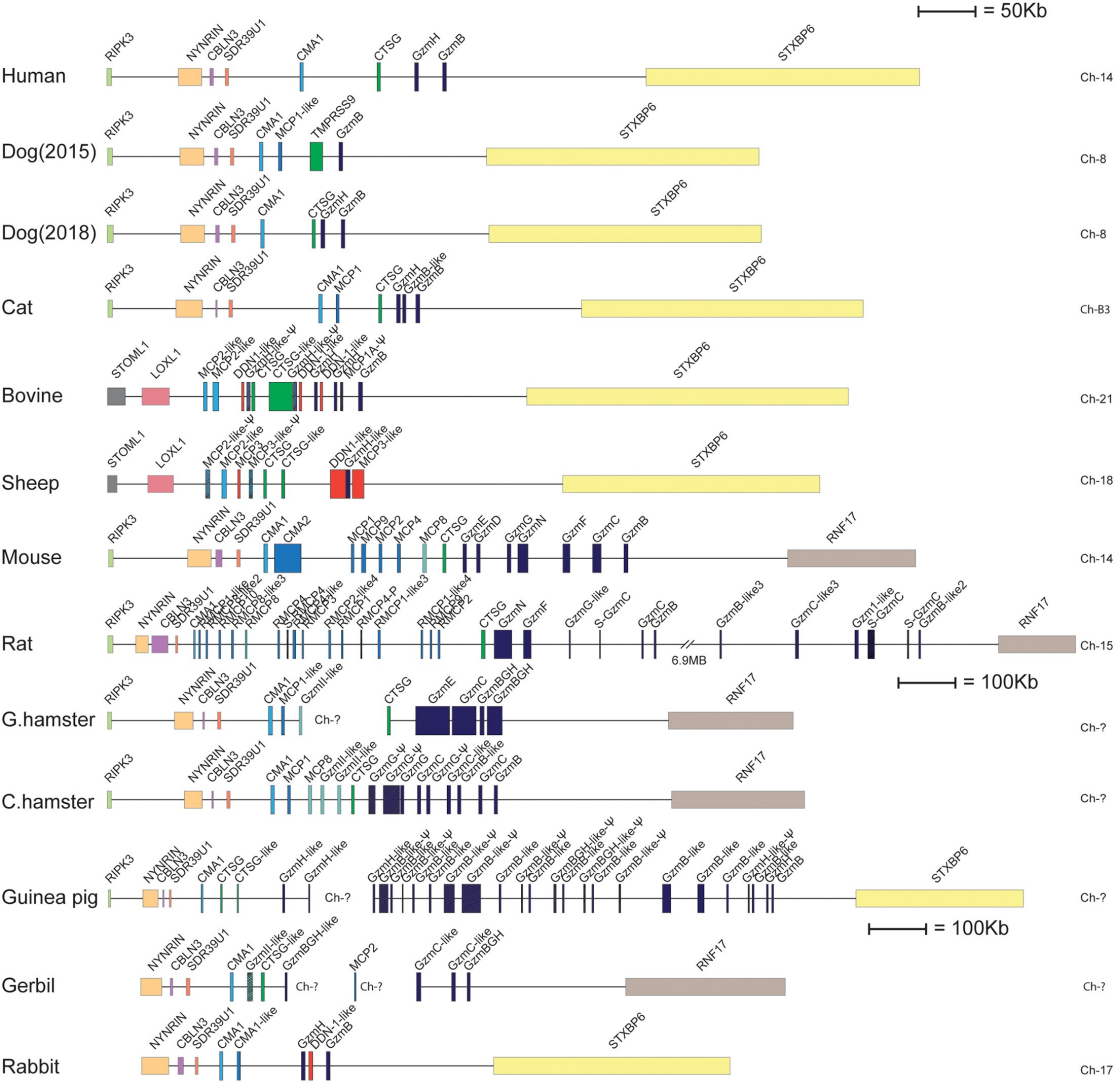
The chymase locus in mammals. A schematic representation in scale of the chymase locus from a panel of placental mammals. This is the locus encoding the α and β-chymases, the mMCP-8 family members, cathepsin G and several of the T cell and NK cell expressed granzymes [6]. The genes are colour coded. Granzymes in dark blue, the α-chymase in light blue, the β-chymases is slightly darker blue, cathepsin G in bright green, the mMCP-8 related proteases in a light blue-green colour and the duodenases of ruminants in red. Bordering genes that help in assigning the locus during genomic screening and for analysis of potential rearrangements in areas close to the locus are depicted in other colours including yellow, light green, grey, purple, pink and orange. The genomic coordinates for the chymase loci presented in this figure are as follows, Human Chr14q12; 24,336,021–24,050,297: Dog—Chr8; 4,305,443–4,877,589: Cat—ChrB3; 76,811,637–77,430,021: Bovine—Chr21; 34,633,172–35,305,932: Sheep—Chr18; 33,488,221–34,143,547: Rabbit—Chr17; 44,437,869–45,028,695: Mouse—Chr14qc3; 55,784,995–56,525,032: Rat—Chr15p13-12; 34,470,796–36,744,750: Golden Hamster—Chr (unknown); 2,447,327–2,618,184 (1): Golden Hamster—Chr (unknown); 12,152,784(RNF17)–12,397,1608 CTSG)(2): Chinese Hamster—Chr (unknown); 451,470(RNF17)–929,914 (RIPK3).

In rodents, the β-chymases have increased in number by local gene duplications and also diversified in their functions. One of them has taken over the role of the α-chymase, another has become the major mucosal mast cell protease, mMCP-1 in the mouse and rMCP-2 in the rat [17, 29]. These new mucosal mast cell specific β-chymases have gained important functions in opening the intestinal mucosa for entry of immune components such as antibodies and complement but also inflammatory cells, primarily eosinophils and neutrophils into the intestinal lumen for combatting intestinal parasites. In both mice and rats a few additional β-chymases are also found: rMCP-3 and -4 in rats and mMCP-2 and mMCP-9 in mice (Fig 11). No specific functions have yet been identified for these proteases. Interestingly, mMCP-2, although relatively highly expressed, seems to have a very narrow substrate binding pocket and no proteolytic activity has been found for this protein [17]. Both rMCP-3 and rMCP-4 are expressed in rat mucosal mast cells, although at low levels [31, 32]. rMCP-4 has also been characterized by phage display and has a relatively strict extended specificity indicating a few well defined targets [33]. However, no targets for this protease have yet been identified. Interestingly, both the golden hamster and the Chinese hamster seem to differ quite extensively from the other rodents by only having one β-chymase, indicating that the massive expansion of the β-chymases seen in rats and mice occurred relatively late (Fig 11). Furthermore this expansion of the β-chymases seems to have been relatively independent events in mice and rats, indicating a strong evolutionary driving force in their expansion [5, 6].

In conclusion, from the analyses of the two mast cell chymases from the golden hamster, the change in primary specificity of the α-chymase occurred relatively early in the rodent branch of the mammalian evolutionary tree. It is apparent that the β-chymase in hamsters has also taken over the primary chymase function following the loss of the chymase activity of the original α-chymase, indicating that this also happened relatively early in rodent evolution. One major difference seen between both the golden and the Chinese hamsters compared to mice and rats, is the expansion of the β-chymases. This expansion seems to have occurred relatively late and independently in mice and rats. This is based on the finding that only one β-chymase gene is present in both Chinese and golden hamsters whereas both mice and rats have a relatively large number of such genes. The positioning of the β-chymase genes relative to each other as well as to other genes within the locus differ significantly between mice and rats [5, 6]. The evolutionary driving force behind this massive expansion is also interesting but remains to be elucidated.

## Experimental procedures

### Phylogenetic tree and sequence alignments

A panel of vertebrate chymase locus encoded genes were analyzed for their sequence relatedness using the MrBase analysis program and the Maximum-likelihood algorithm [6]. The alignment in Fig 10 was performed in the DNAStar program using the Clustaw W algorithm.

### Production and purification of recombinant HAM1, HAM2 and the guinea pig chymase

Chymase sequences were retrieved from SwissProt/TrEMB. The two hamster cDNA sequences were subcloned into a pAcGP67B vector that encodes a secretion signal, ubiquitin and an enterokinase (EK) cleavage sequence immediately before cloning site [15, 16]. The resulting expression vectors were transfected into baculovirus-infected insect cells (High Five) (Invitrogen, Carlsbad, CA). Purification was performed on heparin–Sepharose (GE Healthcare, Piscataway, NJ) and the enzymes were activated by EK cleavage (Roche, Nutley, NJ) [16]. After activation, the second heparin–Sepharose column was used to remove EK and released N-terminal peptides.

Protein purity and concentration was estimated by separation on 12.5% SDS-PAGE gels. Protein samples were mixed with sample buffer, and β-mercaptoethanol was added to a final concentration of 5%. To visualize the protein bands, the gels were stained with colloidal Coomassie Brilliant Blue [34].

### Analysis of primary specificity by cleavage of chromogenic substrates

Enzymatic activity was measured against a panel of chromogenic substrates from Bachem (Bubendorf, Switzerland) and Chromogenix (Mölndal, Sweden). Measurements were performed in 96-well microtiter plates with a substrate concentration of 0.2 mM in 200 μl PBS. Hydrolysis at 37°C was monitored spectrophotometrically at 405 nm for up to 6 hrs in a Versamax microplate reader (Molecular Devices, Sunnyvale, CA).

### Determination of cleavage specificity by phage-displayed nonapeptide library

A library of 5x10^7^ unique phage-displayed nonameric peptides was used to determine the cleavage specificity of the two hamster chymases, as previously described [14, 18, 33]. In these T7 phages, the C-terminus of the capsid protein 10 were manipulated to contain a nine amino acids long random peptide followed by a His_6_-tag [33]. An aliquot of the amplified phages (~10^9^ pfu) were bound to 100 μl Ni-NTA beads by their His_6_-tags for 1 hr at 4°C under gentle agitation. Unbound phages were removed by washing ten times in 1.5 ml 1 M NaCl, 0.1% Tween-20 in PBS, pH 7.2, and two subsequent washes with 1.5 ml PBS. The beads were finally resuspended in 1 ml PBS. Activated HAM1 or HAM2 (~0.1 μg) were added to the resuspended beads and left to digest susceptible phage nonapeptides under gentle agitation at room temperature for 2 hours. PBS without protease was used as control. Phages with a random peptide that was susceptible to protease cleavage were released from the Ni-NTA matrix, and the supernatant containing these phages was recovered. To ensure that all of the released phages were recovered the beads were resuspended in 100 μl PBS (pH 7.2) and the supernatant, after mixing and centrifugation, was added to the first supernatant. To ensure that the His_6_-tags had been hydrolyzed on all phages recovered after protease digestion, 15 μl fresh Ni-NTA agarose beads were added to the combined phage supernatant and the mixture agitated for 15 min followed by centrifugation. A control elution of the phages still bound to the beads, using 100 μl 100 mM imidazole showed that at least 1 x 10^8^ phages were attached to the matrix during each selection. Ten μl of the supernatant containing the released phages was used to determine the amount of phages detached in each round of selection. Dilutions of the supernatant were plated in 2.5 ml 0.6% top agarose containing 300 μl of *E*. *coli* (BLT5615), 100 μl diluted supernatant and 100 μl 100 mM IPTG. The remaining volume of the supernatant was added to a 10 ml culture of BLT5615 (OD ~0.6). The bacteria had 30 min prior to phage addition been induced to produce the T7 phage capsid protein by the addition of 100 μl 100 mM IPTG to the culture. The bacteria lysed approximately 75 min after phage addition. The subsequent lysate was centrifuged to remove cell debris and 500 μl of the phage sub-library was added to 100 μl fresh Ni-NTA beads, to start the next round of selection.

Following five or eight rounds of selection, 120 plaques for each of the two hamster proteases were isolated from LB plates after plating in top agarose. Each phage plaque, corresponding to a phage clone, was dissolved in phage extraction buffer (100 mM NaCl and 6 mM MgSO_4_ in 20 mM Tris-HCl pH 8.0) and vigorously shaken for 30 min in order to extract the phages from the agarose. The phage DNA was then amplified by PCR, using primers flanking the variable region of the gene encoding the modified T7 phage capsid protein. After amplification, the PCR reactions of the 96 clones with the most intense PCR fragments after gel analysis were sent in a 96-well plate to GATC Biotech (Germany) for sequencing.

### Generation of a consensus sequence from sequenced phage inserts

Phage sequences were aligned manually based on the results from the chromogenic substrate analyses indicating their P1 preference. For example, in the case of HAM1, sequences with only one aromatic amino acid were aligned first and sequences with more than one possible cleavage site were then aligned to fit this pattern. Amino acids with similar characteristics were grouped together as follows: aromatic amino acids (Phe, Tyr, Trp); negatively charged amino acids (Asp, Glu); positively charged amino acids (Lys, Arg), small aliphatic amino acids (Gly, Ala); larger aliphatic amino acids (Val, Leu, Ile, Pro), hydrophilic amino acids (Ser, Thr, His, Asn, Gln, Cys, Met). The nomenclature by Schechter and Berger [35] was adopted to designate the amino acids in the substrate cleavage region, where P1-P1' corresponds to the scissile bond.

### Sequence logo

We used web logo3 (http://weblogo.threeplusone.com/) [36] server to create the sequence logo of HAM1 and HAM2 phage display sequences. The parameter used to create a sequence logo are probability units options for Y-axis, stack of consensus sequence in X-axis, composition is an auto, error bars selected and custom scheme colour for amino acids in the sequence logo, KRH (yellow), DE (red), FYW (green), STCMNQ (white) and GAVLIP (blue).

### Generation of recombinant substrates for cleavage specificity analysis

A novel substrate was developed to verify the results obtained from the phage display analyses. Two copies of the *E*. *coli* thioredoxin (trx) gene were inserted in tandem into the pET21 vector for bacterial expression (Fig 6A). In the C-terminal end a His_6_- tag was inserted for purification on Ni^2+^ IMAC columns. In the linker region, between the two trx molecules, the different substrate sequences were inserted by ligating double stranded oligonucleotides into two unique restriction sites, one BamHI and one SalI site (Fig 6A). The sequences of the individual clones were verified after cloning by sequencing of both DNA strains. The plasmids were then transformed into the *E*.*coli* Rosetta gami strain for protein expression (Novagen, Merck, Darmstadt, Germany). A 10 ml overnight culture of the bacteria harbouring the plasmid was diluted 10 times in LB + Amp and grown at 37°C for 1–2 hr until the OD (600 nm) reached 0.5. IPTG was then added to a final concentration of 1 mM. The culture was then grown at 37°C for an additional 3 hr under vigorous shaking, after which the bacteria were pelleted by centrifugation at 3500 rpm for 12 min. The pellet was washed once with 25 ml PBS + 0.05% Tween 20. The pellet was then dissolved in 2 ml PBS and sonicated 6 x 30 sec to open the cells. The lysate was centrifuged at 13000 rpm for 10 min and the supernatant was transferred to a new tube. Ni-NTA slurry (0.5 ml) (50% slurry concentration) (Qiagen, Hilden, Germany) was added and the sample was slowly rotated for 45 min at RT. The sample was then transferred to a 2 ml column and the supernatant was allowed to slowly pass through the filter leaving the Ni-NTA beads with the bound protein in the column. The column was then washed four times with 1 ml of washing buffer (PBS + 0.05% Tween + 10 mM Imidazole + 1 M NaCl). Elution of the protein was performed by adding 150 μl elution buffer followed by five 300 μl fractions of elution buffer (PBS + 0.05% Tween 20 + 100 mM imidazole). Each fraction was collected individually. Samples (10 μl) from each of the eluted fractions were then mixed with 1 volume of 2x sample buffer and 1 μl β-mercaptoethanol and then heated for 3 min at 80°C. The samples were analysed on a SDS bis tris 4–12% PAGE gel and the second and third fractions that contained the most protein were pooled. The protein concentration of the combined fractions was determined by Bio-Rad DC Protein assay (Bio-Rad Laboratories Hercules, CA USA). Approximately 25 μg of recombinant protein was added to each 50 μl cleavage reaction (in PBS). Ten μl from this tube was removed before adding the enzyme, the 0 min time point. The active enzyme was then added (approximately 34 or 20 ng of the HAM1 or 17 ng for the HAM2) and the reaction was kept at room temperature during the entire experiment. Samples (10 μl) were removed at the indicated time points (15 min, 45 min and 150 min) and stopped by addition of one volume of 2x sample buffer. β−mercapto-ethanol (1 μl) was then added to each sample followed by heating for 3 min at 80°C. Twenty μl from each of these samples was then analysed on 4–12% pre-cast SDS-PAGE gels (Invitrogen, Carlsbad, CA, USA). The gels were stained overnight in colloidal Coomassie staining solution and de-stained according to previously described procedures [34].

## Abbreviations

EK: enterokinase
HC: human chymase
MC: mast cell; amino acids, amino acid(s)
mMCP: mouse mast cell protease
Ni-NTA: nickel-nitrilotriacetic acid
rMCP: rat mast cell protease

## References

[1] Galli SJ, Starkl P, Marichal T, Tsai M. Mast cells and IgE in defense against venoms: Possible "good side" of allergy? Allergol Int. 2016;65(1):3–15. 10.1016/j.alit.2015.09.002 . 26666482 10.1016/j.alit.2015.09.002

[2] Hellman LT, Akula S, Thorpe M, Fu Z. Tracing the Origins of IgE, Mast Cells, and Allergies by Studies of Wild Animals. Frontiers in immunology. 2017;8:1749 10.3389/fimmu.2017.01749 . 29312297 10.3389/fimmu.2017.01749 PMC5742104

[3] Pejler G, Ronnberg E, Waern I, Wernersson S. Mast cell proteases: multifaceted regulators of inflammatory disease. Blood. 2010;115(24):4981–90. Epub 2010/03/18. 10.1182/blood-2010-01-257287 . 20233968 10.1182/blood-2010-01-257287

[4] Caughey GH. Mast cell proteases as protective and inflammatory mediators. Adv Exp Med Biol. 2011;716:212–34. 10.1007/978-1-4419-9533-9_12 . 21713659 10.1007/978-1-4419-9533-9_12 PMC3954859

[5] Hellman L, Thorpe M. Granule proteases of hematopoietic cells, a family of versatile inflammatory mediators—an update on their cleavage specificity, in vivo substrates, and evolution. Biol Chem. 2014;395(1):15–49. 10.1515/hsz-2013-0211 . 23969467 10.1515/hsz-2013-0211

[6] Akula S, Thorpe M, Boinapally V, Hellman L. Granule Associated Serine Proteases of Hematopoietic Cells—An Analysis of Their Appearance and Diversification during Vertebrate Evolution. PLoS One. 2015;10(11):e0143091 10.1371/journal.pone.0143091 . 26569620 10.1371/journal.pone.0143091 PMC4646688

[7] Hallgren J, Pejler G. Biology of mast cell tryptase. An inflammatory mediator. Febs J. 2006;273(9):1871–95. 10.1111/j.1742-4658.2006.05211.x . 16640553 10.1111/j.1742-4658.2006.05211.x

[8] Trivedi NN, Tong Q, Raman K, Bhagwandin VJ, Caughey GH. Mast cell alpha and beta tryptases changed rapidly during primate speciation and evolved from gamma-like transmembrane peptidases in ancestral vertebrates. J Immunol. 2007;179(9):6072–9. . 17947681 10.4049/jimmunol.179.9.6072 PMC2366170

[9] Reimer JM, Samollow PB, Hellman L. High degree of conservation of the multigene tryptase locus over the past 150–200 million years of mammalian evolution. Immunogenetics. 2010;62(6):369–82. Epub 2010/04/13. 10.1007/s00251-010-0443-2 . 20383634 10.1007/s00251-010-0443-2

[10] Huang RY, Blom T, Hellman L. Cloning and structural analysis of MMCP-1, MMCP-4 and MMCP-5, three mouse mast cell-specific serine proteases. Eur J Immunol. 1991;21(7):1611–21. 10.1002/eji.1830210706 . 2060576 10.1002/eji.1830210706

[11] Chandrasekharan UM, Sanker S, Glynias MJ, Karnik SS, Husain A. Angiotensin II-forming activity in a reconstructed ancestral chymase. Science. 1996;271(5248):502–5. . 8560264 10.1126/science.271.5248.502

[12] Gallwitz M, Reimer JM, Hellman L. Expansion of the mast cell chymase locus over the past 200 million years of mammalian evolution. Immunogenetics. 2006;58(8):655–69. 10.1007/s00251-006-0126-1 . 16807745 10.1007/s00251-006-0126-1

[13] Kunori Y, Koizumi M, Masegi T, Kasai H, Kawabata H, Yamazaki Y, et al Rodent alpha-chymases are elastase-like proteases. European journal of biochemistry / FEBS. 2002;269(23):5921–30. . 12444981 10.1046/j.1432-1033.2002.03316.x

[14] Karlson U, Pejler G, Tomasini-Johansson B, Hellman L. Extended substrate specificity of rat mast cell protease 5, a rodent alpha-chymase with elastase-like primary specificity. J Biol Chem. 2003;278(41):39625–31. 10.1074/jbc.M301512200 . 12900423 10.1074/jbc.M301512200

[15] Kervinen J, Abad M, Crysler C, Kolpak M, Mahan AD, Masucci JA, et al Structural basis for elastolytic substrate specificity in rodent alpha-chymases. J Biol Chem. 2008;283(1):427–36. 10.1074/jbc.M707157200 . 17981788 10.1074/jbc.M707157200

[16] Kervinen J, Crysler C, Bayoumy S, Abad MC, Spurlino J, Deckman I, et al Potency variation of small-molecule chymase inhibitors across species. Biochem Pharmacol. 2010;80(7):1033–41. Epub 2010/07/06. 10.1016/j.bcp.2010.06.014 . 20599788 10.1016/j.bcp.2010.06.014

[17] Andersson MK, Pemberton AD, Miller HR, Hellman L. Extended cleavage specificity of mMCP-1, the major mucosal mast cell protease in mouse-High specificity indicates high substrate selectivity. Mol Immunol. 2008;45(9):2548–58. 10.1016/j.molimm.2008.01.012 . 18313755 10.1016/j.molimm.2008.01.012

[18] Andersson MK, Karlson U, Hellman L. The extended cleavage specificity of the rodent beta-chymases rMCP-1 and mMCP-4 reveal major functional similarities to the human mast cell chymase. Mol Immunol. 2008;45(3):766–75. 10.1016/j.molimm.2007.06.360 . 17681377 10.1016/j.molimm.2007.06.360

[19] Andersson MK, Enoksson M, Gallwitz M, Hellman L. The extended substrate specificity of the human mast cell chymase reveals a serine protease with well-defined substrate recognition profile. Int Immunol. 2009;21(1):95–104. 10.1093/intimm/dxn128 . 19073880 10.1093/intimm/dxn128

[20] Thorpe M, Yu J, Boinapally V, Ahooghalandari P, Kervinen J, Garavilla LD, et al Extended cleavage specificity of the mast cell chymase from the crab-eating macaque (Macaca fascicularis): an interesting animal model for the analysis of the function of the human mast cell chymase. Int Immunol. 2012;12:771–82. Epub 2012/09/06. 10.1093/intimm/dxs081 . 22949566 10.1093/intimm/dxs081

[21] Reimer JM, Enoksson M, Samollow PB, Hellman L. Extended substrate specificity of opossum chymase-Implications for the origin of mast cell chymases. Mol Immunol. 2008;45(7):2116–25. 10.1016/j.molimm.2007.10.015 . 18022236 10.1016/j.molimm.2007.10.015

[22] Andersson MK, Thorpe M, Hellman L. Arg143 and Lys192 of the human mast cell chymase mediate the preference for acidic amino acids in position P2' of substrates. Febs J. 2010;277(10):2255–67. Epub 2010/04/29. 10.1111/j.1742-4658.2010.07642.x . 20423454 10.1111/j.1742-4658.2010.07642.x

[23] Lutzelschwab C, Huang MR, Kullberg MC, Aveskogh M, Hellman L. Characterization of mouse mast cell protease-8, the first member of a novel subfamily of mouse mast cell serine proteases, distinct from both the classical chymases and tryptases. Eur J Immunol. 1998;28(3):1022–33. 10.1002/(SICI)1521-4141(199803)28:03<1022::AID-IMMU1022>3.0.CO;2-1 . 9541598 10.1002/(SICI)1521-4141(199803)28:03<1022::AID-IMMU1022>3.0.CO;2-1

[24] Poorafshar M, Helmby H, Troye-Blomberg M, Hellman L. MMCP-8, the first lineage-specific differentiation marker for mouse basophils. Elevated numbers of potent IL-4-producing and MMCP-8-positive cells in spleens of malaria-infected mice. Eur J Immunol. 2000;30(9):2660–8. 10.1002/1521-4141(200009)30:9<2660::AID-IMMU2660>3.0.CO;2-I . 11009100 10.1002/1521-4141(200009)30:9<2660::AID-IMMU2660>3.0.CO;2-I

[25] Wada T, Ishiwata K, Koseki H, Ishikura T, Ugajin T, Ohnuma N, et al Selective ablation of basophils in mice reveals their nonredundant role in acquired immunity against ticks. J Clin Invest. 2010;120(8):2867–75. Epub 2010/07/29. 10.1172/JCI42680 . 20664169 10.1172/JCI42680 PMC2912199

[26] Gallwitz M, Enoksson M, Thorpe M, Ge X, Hellman L. The extended substrate recognition profile of the dog mast cell chymase reveals similarities and differences to the human chymase. Int Immunol. 2010;22(6):421–31. Epub 2010/03/27. 10.1093/intimm/dxq021 . 20338912 10.1093/intimm/dxq021

[27] Gallwitz M, Enoksson M, Thorpe M, Hellman L. The extended cleavage specificity of human thrombin. PLoS One. 2012;7(2):e31756 Epub 2012/03/03. 10.1371/journal.pone.0031756 . 22384068 10.1371/journal.pone.0031756 PMC3288055

[28] Chahal G, Thorpe M, Hellman L. The Importance of Exosite Interactions for Substrate Cleavage by Human Thrombin. PLoS One. 2015;10(6):e0129511 10.1371/journal.pone.0129511 . 26110612 10.1371/journal.pone.0129511 PMC4482499

[29] Fu Z, Thorpe M, Hellman L. rMCP-2, the Major Rat Mucosal Mast Cell Protease, an Analysis of Its Extended Cleavage Specificity and Its Potential Role in Regulating Intestinal Permeability by the Cleavage of Cell Adhesion and Junction Proteins. PLoS One. 2015;10(6):e0131720 10.1371/journal.pone.0131720 . 26114959 10.1371/journal.pone.0131720 PMC4482586

[30] Thorpe M, Akula S, Hellman L. Channel catfish granzyme-like I is a highly specific serine protease with metase activity that is expressed by fish NK-like cells. Developmental and comparative immunology. 2016;63:84–95. 10.1016/j.dci.2016.05.013 . 27216028 10.1016/j.dci.2016.05.013

[31] Lutzelschwab C, Pejler G, Aveskogh M, Hellman L. Secretory granule proteases in rat mast cells. Cloning of 10 different serine proteases and a carboxypeptidase A from various rat mast cell populations. J Exp Med. 1997;185(1):13–29. . 8996238 10.1084/jem.185.1.13 PMC2196094

[32] Lutzelschwab C, Lunderius C, Enerback L, Hellman L. A kinetic analysis of the expression of mast cell protease mRNA in the intestines of Nippostrongylus brasiliensis-infected rats. Eur J Immunol. 1998;28(11):3730–7. 10.1002/(SICI)1521-4141(199811)28:11<3730::AID-IMMU3730>3.0.CO;2-0 . 9842915 10.1002/(SICI)1521-4141(199811)28:11<3730::AID-IMMU3730>3.0.CO;2-0

[33] Karlson U, Pejler G, Froman G, Hellman L. Rat mast cell protease 4 is a beta-chymase with unusually stringent substrate recognition profile. J Biol Chem. 2002;277(21):18579–85. 10.1074/jbc.M110356200 . 11896050 10.1074/jbc.M110356200

[34] Neuhoff V, Arold N, Taube D, Ehrhardt W. Improved staining of proteins in polyacrylamide gels including isoelectric focusing gels with clear background at nanogram sensitivity using Coomassie Brilliant Blue G-250 and R-250. Electrophoresis. 1988;9(6):255–62. 10.1002/elps.1150090603 . 2466658 10.1002/elps.1150090603

[35] Schechter I, Berger A. On the size of the active site in proteases. I. Papain. Biochem Biophys Res Commun. 1967;27(2):157–62. . 6035483 10.1016/s0006-291x(67)80055-x

[36] Crooks GE, Hon G, Chandonia JM, Brenner SE. WebLogo: a sequence logo generator. Genome research. 2004;14(6):1188–90. 10.1101/gr.849004 . 15173120 10.1101/gr.849004 PMC419797

